# Arabidopsis PCNAs form complexes with selected D-type cyclins

**DOI:** 10.3389/fpls.2015.00516

**Published:** 2015-07-17

**Authors:** Wojciech K. Strzalka, Chhavi Aggarwal, Weronika Krzeszowiec, Agata Jakubowska, Olga Sztatelman, Agnieszka K. Banas

**Affiliations:** ^1^Department of Plant Biotechnology, Faculty of Biochemistry, Biophysics and Biotechnology, Jagiellonian UniversityKrakow, Poland; ^2^The Bioremediation Department, Malopolska Centre of Biotechnology, Jagiellonian UniversityKrakow, Poland; ^3^Department of Gene Expression, Faculty of Biology, Adam Mickiewicz UniversityPoznan, Poland

**Keywords:** Arabidopsis, PCNA, D-type cyclins, DNA replication, DNA repair, cell cycle

## Abstract

Proliferating Cell Nuclear Antigen (PCNA) is a key nuclear protein of eukaryotic cells. It has been shown to form complexes with cyclin dependent kinases, cyclin dependent kinase inhibitors and the D-type cyclins which are involved in the cell cycle control. In Arabidopsis two genes coding for PCNA1 and PCNA2 proteins have been identified. In this study by analyzing Arabidopsis PCNA/CycD complexes we tested the possible functional differentiation of PCNA1/2 proteins in cell cycle control. Most out of the 10 cyclins investigated showed only nuclear localization except CycD2;1, CycD4;1, and CycD4;2 which were observed both in the nucleus and cytoplasm. Using the Y2H, BiFC and FLIM-FRET techniques we identified D-type cyclins which formed complexes with either PCNA1 or PCNA2. Among the candidates tested only CycD1;1, CycD3;1, and CycD3;3 were not detected in a complex with the PCNA proteins. Moreover, our results indicate that the formation of CycD3;2/PCNA and CycD4;1/PCNA complexes can be regulated by other as yet unidentified factor(s). Additionally, FLIM-FRET analyses suggested that *in planta* the distance between PCNA1/CycD4;1, PCNA1/CycD6;1, PCNA1/CycD7;1, and PCNA2/CycD4;2 proteins was shorter than that between PCNA2/CycD4;1, PCNA2/CycD6;1, PCNA2/CycD7;1, and PCNA1/CycD4;2 pairs. These data indicate that the nine amino acid differences between PCNA1 and PCNA2 have an impact on the architecture of Arabidopsis CycD/PCNA complexes.

## Introduction

Proliferating Cell Nuclear Antigen (PCNA) is the fundamental eukaryotic protein which is present mainly in the nuclei of dividing cells. Its elevated synthesis is observed in the early S phase of the cell cycle (Morris and Mathews, 1989). Molecular studies on plant organisms have demonstrated that the genomes of some species, e.g., carrot (Hata et al., 1992), maize (Lopez et al., 1997) and Arabidopsis (Arabidopsis Genome Initiative, 2000) have two genes coding for PCNA1 and PCNA2 proteins. An analysis of PCNA amino acid sequences from different plant species, including rice (Suzuka et al., 1991), maize (Lopez et al., 1997), common bean (Strzalka and Ziemienowicz, 2007), and runner bean (Strzalka et al., 2010) showed that the identity between these proteins is over 85%. Interestingly, although the amino acid sequence identity between Arabidopsis/yeast and Arabidopsis/human PCNA is 40 and 65% respectively, crystallographic data demonstrated that these proteins have a very similar and conserved three dimensional architecture (Gulbis et al., 1996; Strzalka et al., 2009). A PCNA monomer, a 29 kDa polypeptide composed of two structural domains linked by an inter-domain connecting loop, naturally forms a homotrimer, ring-like in structure (Strzalka and Ziemienowicz, 2011). This trimer plays a crucial function during DNA replication. After the initiation of DNA synthesis to provide the undisturbed continuation of this process, PCNA must be recruited to the replication fork. With the help of Replication Factor C, PCNA is loaded onto the DNA duplex where it acts as a sliding platform which coordinates and affects the activity of the proteins involved in DNA replication (Wu et al., 1996; Tom et al., 2001; Strzalka and Ziemienowicz, 2011). PCNA is involved not only in DNA replication but also in DNA repair and cell cycle control (Strzalka and Ziemienowicz, 2011). In yeasts, animals and plants, cell cycle proteins, cyclin dependent kinases (CDK), cyclin dependent kinase inhibitors (CDKI) and D-type cyclins were found in complexes with PCNA (Paz Sanchez et al., 2002; Vivona and Kelman, 2003). Detailed studies on mouse cyclin D1, D3, and PCNA revealed that both the N- and C-terminal regions of PCNA are involved in interactions with these cyclins (Matsuoka et al., 1994).

Comparative analysis of mammalian and higher plant genomes has demonstrated that the latter have a much higher number of genes coding for D-type cyclins. For example, in contrast to the human genome which encodes only cyclin D1, D2, and D3 (Sherr and Roberts, 2004), the genome of Arabidopsis, rice and maize has 10, 14, and 17 genes respectively coding for D-type cyclins. These are grouped into seven different classes (Arabidopsis Genome Initiative, 2000; Buendia-Monreal et al., 2011). In the sequence of plant D-type cyclins, typical motifs and domains which are also characteristic of other cyclin types can be distinguished. These include, (i) a cyclin core composed of either a conserved N-terminal domain or both an N- and less conserved C-terminal domain, and (ii) a cyclin box located within the N-terminal domain which is a binding site for CDKs. (Wang et al., 2004; Buendia-Monreal et al., 2011). Additionally, in some of the D-type cyclins there is, (i) a PEST domain, rich in proline, glutamate, serine and threonine residues which is a marker for unstable proteins (Wang et al., 2004; Buendia-Monreal et al., 2011), and (ii) a conserved retinoblastoma protein (pRB) binding motif located at the N-terminus (Buendia-Monreal et al., 2011). The consensus sequence of the pRB binding motif is LXCXE where L, E, C represent leucin, cysteine and glutamic acid respectively, while X represents any amino acid residue.

D-type cyclins interact with CDKs regulating their activity. They can be found in complexes not only with CDKs but also other proteins. For example, the human CycD1 was found to be a component of a larger complex containing CDK2, CDK4, CDK5, p21, and PCNA (Xiong et al., 1992). Moreover, the results from studies on mammalian cell cycle proteins showed that the excess of CycD1 repressed cell proliferation by inhibiting DNA synthesis and CDK2 activity, possibly through the binding of CycD1 to PCNA and CDK2 (Fukami-Kobayashi and Mitsui, 1999). The mammalian cyclins D1, D2, D3 and their partners CDK4 and CDK6 have been shown to act early in G1 phase (Sherr and Roberts, 2004). It is assumed that CycD/CDK complexes bind and phosphorylate pRB in the early G1 phase. This results in the release of the E2F transcription factor and allows cells to progress from the G1 to the S phase (Harbour and Dean, 2000). Studies on plant cyclins have revealed that, as with yeast and mammalian proteins they can form complexes with CDKs. Co-immunoprecipitation experiments demonstrated that Arabidopsis CycD4;2 can be observed in complex with CDKA;1, CDKB1;1, or CDKB2;1 (Kono et al., 2006). A constructed Arabidopsis protein interaction network revealed the presence of D-type cyclins/CDK complexes (Boruc et al., 2010). Another study showed the formation of CycD2;2/CDKA, CycD2;2/CDKB1;1, CycD4;2/CDKA, CycD4;1/CDKB1;1, CycD5;3/CDKA, or CycD5;2/CDKB1;1 complexes in germinating maize. Additionally, changes in the total level of the tested (i) D-type cyclins, (ii) CDKs and iii) both D-type cyclins and CDKs present in the CycD/CDK complexes were also demonstrated (Godinez-Palma et al., 2013). The analysis of the tobacco retinoblastoma-related (RBR) protein and CycD3;1 using Y2H and pull down/immunoprecipitation experiments revealed that these proteins are parts of the same complex (Nakagami et al., 1999). Moreover, in this study the tobacco Cdc2/CycD3;1 complex, produced and purified from the insect cells, has been shown to phosphorylate RBR protein. Similarly, Arabidopsis CycD2;1 and CDKA;1 were shown to form complex with maize RBR protein (Boniotti and Gutierrez, 2001). Concluding, the presence of CycD/CDK as well as pRB/E2F complexes in plant cells suggests that the mitogenic signal transmission pathway is conserved in higher eukaryotes (Meijer and Murray, 2000).

Despite significant progress in plant D-type cyclin studies over recent years, the role of these proteins in Arabidopsis cells in the context of interaction with PCNA is still unknown. To shed more light on the interplay between Arabidopsis PCNA1/2 proteins and D-type cyclins we employed the following techniques: a split ubiquitin yeast two hybrid system (Y2H), a bimolecular fluorescence complementation (BiFC) and fluorescence-lifetime imaging microscopy-Froster resonance energy transfer (FLIM-FRET). In this study we present the results from the analysis of Arabidopsis CycD/PCNA complexes.

## Materials and methods

### Computational analysis

The nucleotide and protein sequences of Arabidopsis D-type cyclins were identified using the NCBI database. The N- and C-terminal cyclin domains were detected with the help of the Pfam database (http://pfam.xfam.org/). The putative PEST motif analysis was performed using pestfind software (http://emboss.bioinformatics.nl/cgi-bin/emboss/pestfind) with *E*-value 0.01 as the cutoff. The theoretical pI and MW values were calculated through the ExPASy bioinformatics resource portal. The exon-intron gene structure was built with the help of exon-intron graphic maker (http://wormweb.org/exonintron). The presence of the putative importin α-dependent nuclear localization signal in Arabidopsis D-type cyclins was analyzed using cNLS mapper (http://nls-mapper.iab.keio.ac.jp/cgi-bin/NLS_Mapper_form.cgi) with the cut off 6.0.

### Construction of vectors used for Y2H analysis

The pDHB1 bait and pPR3-N prey plasmids used in the split-ubiquitin Y2H system transactivating starter kit (MoBiTec) were reconstructed into Gateway-compatible vectors. The gateway cassette containing the *ccdB* gene was amplified using a RAPID PCR mix (A&A Biotechnology, Poland), containing an appropriate set of primers (Supplementary Table 1), with pDONR221 as a template. The PCR product, pDHB1 and pPR3-N vectors were digested with the SfiI restriction enzyme (FastDigest, Thermo Scientific). The digested PCR product was ligated into both plasmids and transformed into the *E. coli* DB3.1 strain. The bacterial colonies selected on an LB plate supplemented with 25 mg/L of chloramphenicol were used for the isolation of the pDHB1Gateway and pPR3-NGateway vectors. Then, the kanamycin resistance coding gene (*KanR*) of the pDHB1Gateway vector was replaced with a spectinomycin resistance coding gene (*SpmR*). The *SpmR* gene was amplified from the pK7WGF2 vector using Easy-A polymerase (Stratagene) and an appropriate set of primers (Supplementary Table 1). For gene exchange, yeast homologous recombination was employed. The NMY51 strain cells were transformed with a mixture containing pDHB1Gateway vector digested with HindIII and XhoI and the PCR product followed by selection on an SD-Leu solid medium. The plasmids isolated from growing yeast colonies were transformed into the DB3.1 cells. Transformed bacterial cells were plated on an LB solid medium supplemented with spectinomycin (100 mg/L) and the plasmid from growing colonies was isolated.

To construct the entry vectors coding for Arabidopsis D-type cyclins, PCNA1 and PCNA2 with stop codon appropriate open reading frames (ORFs) were amplified with the help of Pfu polymerase (Fermentas) using specific primers (Supplementary Table 1). The PCR products were purified and cloned into the pDONR221 vector using a Gateway BP Clonase II Enzyme mix (Life Technologies) followed by sequencing. The other entry pDONR221 plasmids containing ORFs without stop codon were either purchased from the Arabidopsis Biological Stock Centre (ABRC) or constructed as previously described (Strzalka et al., 2012). To prepare the destination vectors appropriate ORFs were transferred from pDONR221 either into the pDHB1Gateway (bait) or pPR3-NGateway (prey) vector (Supplementary Table 2) with the help of a Gateway LR Clonase enzyme mix (Life Technologies).

### Construction of vectors used for plant transformation

To prepare final binary vectors, pDONR221 plasmids containing appropriate ORFs were either (i) purchased from the ABRC, or (ii) obtained in a previous study (Strzalka et al., 2012), or (iii) constructed in this study (Section Construction of Vectors Used for Y2H Analysis). The ORFs were transferred to destination vectors (Supplementary Table 2) as described in the Section Construction of Vectors Used for Y2H Analysis. The final destination plasmids were transformed into *Agrobacterium tumefaciens* strain C58. The binary vectors containing Arabidopsis PCNA1_GFP, PCNA1_NtermGFP, PCNA1_CtermGFP, PCNA2_GFP, PCNA2_NtermGFP, PCNA2_CtermGFP ORFs were constructed during previous studies (Strzalka et al., 2012, 2013) (Supplementary Table 2).

### Yeast two-hybrid analysis

A split-ubiquitin Y2H system transactivating starter kit was used to test interactions between Arabidopsis D-type cyclins and PCNA1 or PCNA2. Yeast strain NMY51 was transformed with appropriate combinations of bait (pDHB1Gateway) and prey (pPR3-NGateway) plasmids (Supplementary Table 2) along with positive and negative control vectors according to the supplied protocol. After transformation, the yeast cells were transferred onto SC-Leu-Trp selection plates followed by a 3-day incubation at 30°C. The transformed cells were inoculated in a liquid SC-Leu-Trp medium and grown with vigorous shaking overnight at 30°C. The overnight cultures were plated on an SC-Leu-Trp solid medium, an SC-Leu-Trp-His selection solid medium supplemented with 10 mM 3-aminotriazol (3-AT) or a nitrocellulose filter placed on the surface of a YPAD solid medium. The SC plates were incubated for 4 days at 30°C before analysis. The yeast cells plated on nitrocellulose filter/YPAD medium were incubated for 24 h at 30°C. The filter was then immersed in liquid nitrogen for 60 s and placed on Whatman filter paper saturated with buffer A (60 mM Na_2_HPO_4_, 40 mM Na_2_HPO_4_, 10 mM KCl, 1 mM MgSO_4_, 85 mM 2-mercaptoethanol, 1 mg/ml of 5-bromo-4-chloro-3-indolyl-D-galactopyranoside (X-gal), pH 7.0) and kept at 37°C for 18 h.

### Bimolecular fluorescence complementation analysis

Wild type *Nicotiana benthamiana* plants were grown in the greenhouse under natural light supplemented with artificial light (High Pressure Sodium Lamp 600 Watt, Phytolit™) to maintain a 16 h L/8 h D photoperiod at 23°C and relative humidity 40%. For the experiments the leaves of an 8-week old plant were used. The BiFC analysis was performed as described previously (Strzalka et al., 2012, 2013). The interactions were tested using: NtermGFP_PCNA/CycD_CtermGFP/p19 (viral-encoded suppressor of gene silencing), PCNA_NtermGFP/CycD_CtermGFP/p19, CtermGFP_PCNA/CycD_NtermGFP/p19, or PCNA_CtermGFP/CycD_NtermGFP/p19 binary vectors (Supplementary Figure 1). Before imaging the leaves were syringe-infiltrated with water and evaluated with the help of a BioRad MRC 1024 confocal microscope (BioRad Hercules, CA, U.S.A). Images were collected using a 60x (NA 1.4) PlanApo oil-immersion objective mounted on the microscope. The excitation wavelength was 488 nm emitted by a 100 mW argon-ion laser (ITL. U.S.A.). GFP fluorescence was collected with a 540 DF30 filter and chloroplast autofluorescence with a 585LP filter.

### FLIM-FRET analysis

The leaves of *N. benthamiana* plants were transiently transformed as described in Section Bimolecular Fluorescence Complementation Analysis using appropriate RFP_PCNA/CycD_GFP and PCNA_RFP/CycD_GFP combinations. Prior to FLIM data collection, the GFP and RFP fluorescence levels in the plant samples within the region of interest were confirmed using a Nikon A1R confocal microscope with excitation at 488 and 543 nm, respectively. FLIM was performed using the Picoquant PicoHarp TCSPC Module. As control of donor-acceptor pairs, GFP (from pK7WGF2) and RFP (from pSITE4CA) were chosen. The GFP (donor) was excited with a 485 nm pulsed diode laser (PDL 800-D, 40 mHz). The excitation light was directly coupled with the microscope and focused on the sample using a CFI Apo 40X water immersion objective lens. GFP emission was selected using a 520/535 nm filter. Photons were detected using a SPAD detector module. Images were acquired with a frame size of 256 × 256 pixels. Data analysis was performed with Picoquant's Symphotime software. From the obtained images, complete fluorescence lifetime decays were calculated per pixel for nuclei and fitted using a double exponential decay model. χ 2 of 1 was considered as a perfect fit. For FRET analysis the fluorescence lifetime of the donor/acceptor pair (τ DA) was compared with that of donor alone (τ D). The FRET efficiency (E) was calculated as E = (1−τ DA/τ D) × 100%, where τ D is the fluorescence lifetime of a donor in the absence of an acceptor and τ DA that of a donor in the presence of an acceptor. At least six to eight nuclei per combination were analyzed and the average of the values was taken for analysis.

## Results

### In silico analysis of arabidopsis D-type cyclins

**Figure 1:**
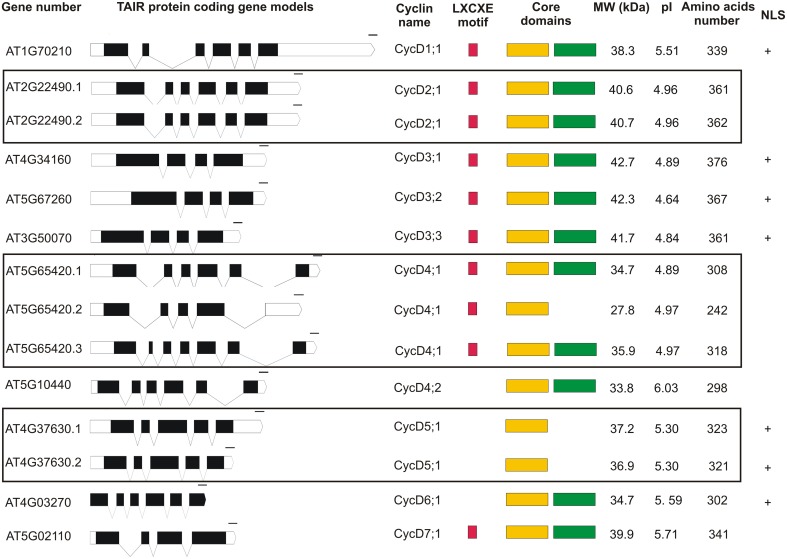
**The genomic and protein organization of Arabidopsis D-type cyclins**. The white and black bars represent the untranslated (UTRs) and coding region respectively. The regions within slanting lines represent introns. The red, yellow and green colors represent the retinoblastoma protein binding motif (LXCXE), cyclin N- and C-terminal domain respectively. (+) represents the presence of the putative importin-α nuclear localization signal.

Analysis of the *Arabidopsis thaliana* genome database revealed that 10 D-type cyclins are encoded by the nuclear DNA of this plant (Figure 1). The data deposited in TAIR (The Arabidopsis Information Resource http://www.arabidopsis.org) show only one type of gene model for CycD1;1, CycD3;1, CycD3;2, CycD3;3, CycD4;2, CycD6;1, CycD7;1, two for CycD2;1, CycD5;1 and three for CycD4;1. Transcript coding for CycD1;1, CycD2;1 (AT2G22490.1 and AT2G22490.2 splicing variants), CycD4;1 (splicing variant AT5G65420.1), CycD4;2 and CycD6;1 consist of six exons. In the structure of gene coding for CycD3;1, CycD3;2, CycD3;3 and CycD7;1 four exons are found. The CycD4;1 (transcript variant AT5G65420.2) and CycD5;1 (AT4G37630.1 and AT4G37630.2 transcript variants) are products of five exons. Finally, transcript variant AT5G65420.3 coding for CycD4;1 is a product of seven exons. The gene coding for CycD1;1, CycD2;1, CycD3;1/CycD5;1/CycD6;1, CycD3;2/CycD4;1/CyD4;2/CycD7;1, CycD3;3 are located on chromosome 1, 2, 4, 5 and 3 respectively. Arabidopsis D-type cyclins are acidic proteins with an isoelectric point ranging from 4.64 to 6.03 and a molecular weight (MW) between 27.8 and 42.7 kDa. Within the first thirty amino acids of CycD1;1, CycD2;1, CycD3;1, CycD3;2, CycD3;3, CycD4;1, and CycD7;1, a consensus pRB binding sequence (LXCXE motif) is found (Figure 1). The analysis of the investigated D-type cyclin amino acid sequences using the Pfam database revealed that the cyclin core of CycD1;1, CycD2;1 (both splicing variants), CycD3;1, CycD3;2, CycD3;3, CycD4;1 (AT5G65420.1 and AT5G65420.3 gene models), CycD4;2, CycD6;1 and CycD7;1 is composed of a conserved N- and less conserved C-terminal domain. On the other hand for CycD4;1 (AT5G65420.2 splicing variant) and CycD5;1 (AT4G37630.1 and AT4G37630.2 transcript variants) only an N-terminal domain fold was detected (Figure 1). The putative PEST motif was identified in all of the analyzed Arabidopsis D-type cyclins. Finally, using cNLS mapper tool the presence of putative importin α-dependent nuclear localization signal was predicted for CycD1;1, CycD3;1, CycD3;2, CycD3;3, CycD5;1 (both splicing variants) and CycD6;1 (Figure 1).

### Analysis of subcellular localization of arabidopsis D-type cyclins

**Figure 2:**
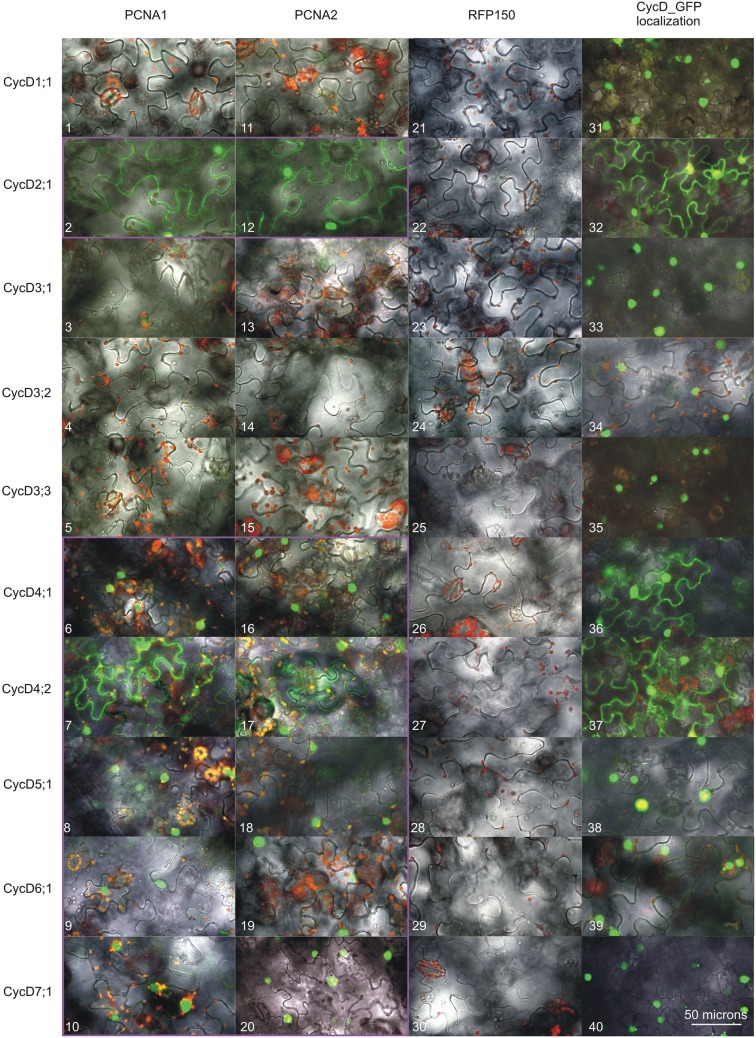
**Analysis of D-type cyclins subcellular localization and the formation of complexes with either PCNA1 or PCNA2**. Confocal images of *N. benthamiana* leaf cells expressing transiently analyzed open reading frames. Split GFP complex formed between PCNA1_CtermGFP and (1) CycD1;1_NtermGFP, (2) CycD2;1_NtermGFP, (3) CycD3;1_NtermGFP, (4) CycD3;2_NtermGFP, (5) CycD3;3_NtermGFP, (6) CycD4;1_NtermGFP, (7) CycD4;2_NtermGFP, (8) CycD5;1_NtermGFP, (9) CycD6;1_NtermGFP, (10) CycD7;1_NtermGFP. Split GFP complex formed between PCNA2_CtermGFP and (11) CycD1;1_NtermGFP, (12) CycD2;1_NtermGFP, (13) CycD3;1_NtermGFP, (14) CycD3;2_NtermGFP, (15) CycD3;3_NtermGFP, (16) CycD4;1_NtermGFP, (17) CycD4;2_NtermGFP, (18) CycD5;1_NtermGFP, (19) CycD6;1_NtermGFP, (20) CycD7;1_NtermGFP. Split GFP complex formed between RFP150_CtermGFP and (21) CycD1;1_NtermGFP, (22) CycD2;1_NtermGFP, (23) CycD3;1_NtermGFP, (24) CycD3;2_NtermGFP, (25) CycD3;3_NtermGFP, (26) CycD4;1_NtermGFP, (27) CycD4;2_NtermGFP, (28) CycD5;1_NtermGFP, (29) CycD6;1_NtermGFP, (30) CycD7;1_NtermGFP. (31) CycD1;1_GFP, (32) CycD2;1_GFP, (33) CycD3;1_GFP, (34) CycD3;2_GFP, (35) CycD3;3_GFP, (36) CycD4;1_GFP, (37) CycD4;2_GFP, (38) CycD5;1_GFP, (39) CycD6;1_GFP, (40) CycD7;1_GFP. All the images are overlays of the bright field, autofluorescence of chlorophyll (red) and GFP fluorescence (green). The PCNA/CycD complexes are in the magenta frame. This result is representative of three independently repeated experiments.

A subcellular localization analysis of Arabidopsis D-type cyclins_GFP fusions in *N. benthamiana* cells revealed that all of the tested proteins were present in the nucleus (Figure 2). Furthermore, investigation of CycD2;1, Cyc4;1, and CycD4;2 showed that these proteins could be detected not only in the nucleus but also in the cytoplasm (Figure 2), similar to previously tested PCNA1 and PCNA2 (Strzalka et al., 2012).

### Identification of those arabidopsis D-type cyclin candidates which form complexes with PCNA1/2

**Figure 3:**
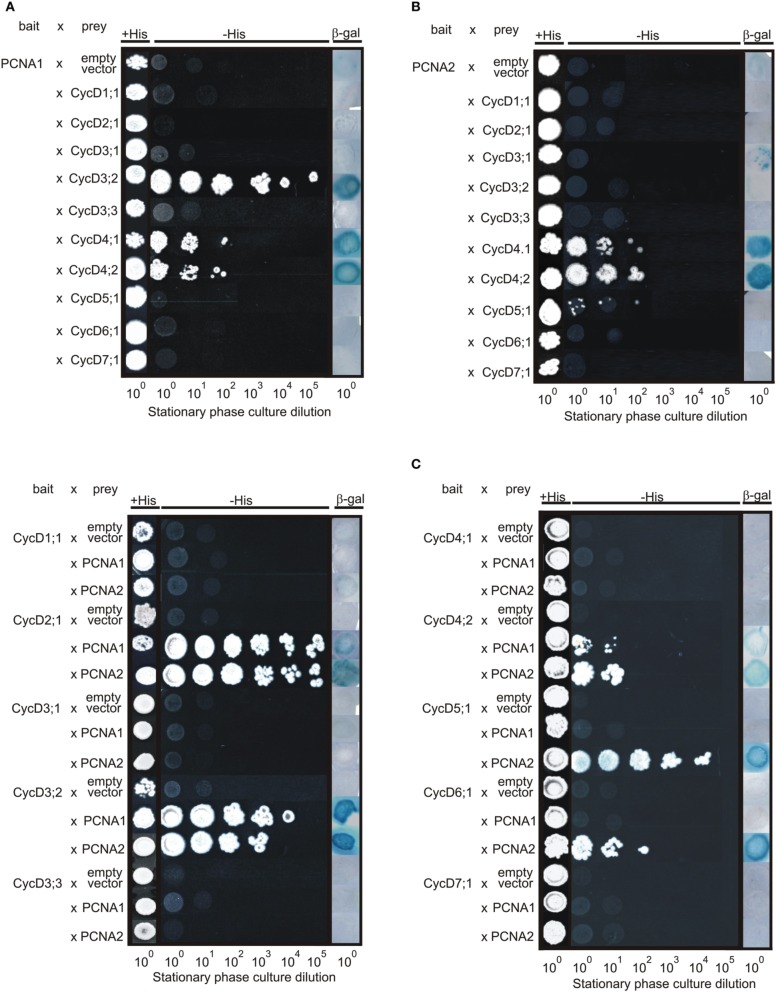
**Analysis of interactions between Arabidopsis PCNA1/2 and D-type cyclins using the split-uiquitin Y2H system**. The interactions were tested in the following combinations. **(A)** PCNA1 (bait)/D-type cyclins (prey), **(B)** PCNA2 (bait)/D-type cyclins (prey), and **(C)** D-type cyclins (bait)/PCNA1/2 (prey). The transformed yeast cells were plated on either an SC-Leu-Trp control solid medium or an SC-Leu-Trp-His selection solid medium supplemented with 10 mM 3-aminotriazol (3-AT). For the beta-galactosidase assay, the yeast transformants were grown on a nitrocellulose filter placed on the surface of a YPAD solid medium followed by incubation with X-gal. The results are representative of three independently repeated experiments.

At the first stage of this study the Y2H technique was employed to identify which Arabidopsis D-type cyclin candidates may form complexes with PCNA1 and/or PCNA2. The interactions between PCNA1/2 and D-type cyclins were tested in two different combinations. In the first combination PCNA1/2 proteins were used as bait. The result of this analysis demonstrated that PCNA1 formed a complex with CycD3;2, CycD4;1, and CycD4;2 (Figure 3A) while PCNA2 showed interaction only with CycD4;1 and CycD4;2 but not CycD3;2 (Figure 3B). Testing the opposite combination, where D-type cyclins were expressed as bait, interactions between CycD2;1/PCNA1, CycD3;2/PCNA1, CycD4;2/PCNA1, CycD2;1/PCNA2, CycD3;2/PCNA2, and CycD4;2/PCNA2 were observed (Figure 3C). Moreover, complex formation was also observed for CycD5;1/PCNA2 and CycD6;1/PCNA2 (Figure 3C).

Following the Y2H analysis, an *N. benthamiana* transient transformation assay was employed. Complex formation between Arabidopsis PCNA1/2 and D-type cyclins was analyzed *in planta* using BiFC and FLIM-FRET techniques. In BiFC studies, four different combinations including CycD_NtermGFP/PCNA_CtermGFP (Figure 2) were analyzed. When CycD1;1, CycD3;1, CycD3;2, and CycD3;3 were investigated, no complexes with PCNA1/2 were observed. On the other hand, presence of GFP fluorescence was observed only in the nucleus when the formation of complexes between PCNA1/2 and CycD4;1, CycD5;1, CycD6;1, or CycD7;1 was tested. Moreover, in the case of CycD2;1/PCNA and CycD4;2/PCNA, complexes were observed both in the nucleus and cytoplasm. Simultaneously to BiFC experiments, FLIM-FRET analysis was performed. First, either GFP alone or GFP together with RFP was transiently expressed in *N. benthamiana* leaf cells using the agroinfiltration method. The lifetime of GFP (donor protein) was determined in the absence (τ D) and in the presence (τ DA) of RFP (potential acceptor), to calculate the value of FRET efficiency for non-interacting GFP/RFP partners (negative control). In the presence of RFP the average lifetime of GFP decreased from 2.51 to 2.48 ns and the calculated FRET efficiency was 1.19% (Table 1) which was used as a threshold. In subsequent experiments the values of FRET efficiency above the threshold were considered as putative PCNA/D-type cyclin complexes. Additionally, a similar experiment was performed for PCNA2_GFP/RFP_PCNA2 pair (positive control) based on the previous studies where the formation of complexes between PCNA2 monomers was shown (Strzalka and Aggarwal, 2013). The calculated FRET efficiency for this pair of proteins was 4.78%. Finally, complex formation between D-type cyclins_GFP and RFP_PCNA1/2 fusions was tested. The FLIM analysis showed a decrease in donor fluorescence lifetime for cyclin D4;1, D4;2, D6;1, and D7;1 when expressed with PCNA1/2 suggesting complex formation between these protein pairs (Table 1). During FLIM measurement, co-localization studies of PCNA1/2 and D-type cyclins (Pearson's correlation coefficient, Table 1) were performed. Despite good co-localization between PCNA1/2 and CycD1;1, CycD2;1, CycD3;1, CycD3;2, CycD3;3, or CycD5;1 the calculated FRET efficiency values were similar or lower than the threshold value, indicating that these protein pairs were not a part of the same complex (Table 1). Interestingly, the value of FRET efficiency obtained for PCNA1/CycD4;1, PCNA1/CycD6;1. PCNA1/CycD7;1, and PCNA2/CycD4;2 pairs was higher than that of PCNA2/CycD4;1, PCNA2/CycD6;1, PCNA2/CycD7;1, and PCNA1/CycD4;2 complexes (Table 1). FLIM-FRET analysis of D-type cyclins_GFP and PCNA1/2_RFP did not demonstrate formation of complexes between tested proteins (Table 1).

**Table 1:** **Results of FLIM-FRET analysis and co-localization measurement (Pearson's correlation coefficient)**.

**Donor protein**	**Acceptor protein**	**Pearson's correlation coefficient ± SD**	**Donor lifetime [τD (ns) ± SD]**	**Donor lifetime in the presence of potential acceptor [τ DA (ns) ± SD]**	**FRET efficiency (%)**
GFP	RFP	0.78 ± 0.03	2.51 ± 0.01	2.48 ± 0.01	1.19
PCNA2_GFP	RFP_PCNA2	0.77 ± 0.01	2.51 ± 0.01	2.39 ± 0.01	4.78
CycD1;1_GFP	RFP_PCNA1	0.83 ± 0.01	2.38 ± 0.02	2.37 ± 0.02	0.42
CycD1;1_GFP	RFP_PCNA2	0.84 ± 0.01	2.38 ± 0.02	2.36 ± 0.01	0.84
CycD2;1_GFP	RFP_PCNA1	0.71 ± 0.09	2.45 ± 0.01	2.41 ± 0.01	1.63
CycD2;1_GFP	RFP_PCNA2	0.70 ± 0.04	2.45 ± 0.01	2.43 ± 0.02	0.82
CycD3;1_GFP	RFP_PCNA1	0.92 ± 0.02	2.45 ± 0.02	2.44 ± 0.02	0.41
CycD3;1_GFP	RFP_PCNA2	0.92 ± 0.01	2.45 ± 0.02	2.44 ± 0.03	0.41
CycD3;2_GFP	RFP_PCNA1	0.86 ± 0.01	2.49 ± 0.01	2.48 ± 0.00	0.40
CycD3;2_GFP	RFP_PCNA2	0.84 ± 0.05	2.49 ± 0.01	2.48 ± 0.01	0.40
CycD3;3_GFP	RFP_PCNA1	0.80 ± 0.02	2.43 ± 0.04	2.42 ± 0.02	0.41
CycD3;3_GFP	RFP_PCNA2	0.90 ± 0.02	2.43 ± 0.04	2.40 ± 0.02	1.23
CycD4;1_GFP	RFP_PCNA1	0.80 ± 0.01	2.53 ± 0.01	2.43 ± 0.02	3.95
CycD4;1_GFP	RFP_PCNA2	0.71 ± 0.02	2.53 ± 0.01	2.48 ± 0.02	1.98
CycD4;2_GFP	RFP_PCNA1	0.70 ± 0.00	2.53 ± 0.01	2.44 ± 0.02	3.56
CycD4;2_GFP	RFP_PCNA2	0.74 ± 0.01	2.53 ± 0.01	2.40 ± 0.01	5.14
CycD5;1_GFP	RFP_PCNA1	0.83 ± 0.01	2.32 ± 0.02	2.31 ± 0.01	0.43
CycD5;1_GFP	RFP_PCNA2	0.85 ± 0.02	2.32 ± 0.02	2.30 ± 0.03	0.86
CycD6;1_GFP	RFP_PCNA1	0.85 ± 0.03	2.44 ± 0.01	2.34 ± 0.02	4.10
CycD6;1_GFP	RFP_PCNA2	0.82 ± 0.04	2.44 ± 0.01	2.37 ± 0.02	2.87
CycD7;1_GFP	RFP_PCNA1	0.93 ± 0.00	2.40 ± 0.01	2.25 ± 0.02	6.25
CycD7;1_GFP	RFP_PCNA2	0.86 ± 0.01	2.40 ± 0.01	2.31 ± 0.02	3.75
CycD1;1_GFP	PCNA1_RFP	0.87 ± 0.02	2.48 ± 0.01	2.45 ± 0.02	1.13
CycD1;1_GFP	PCNA2_RFP	0.87 ± 0.01	2.48 ± 0.01	2.49 ± 0.02	0.08
CycD2;1_GFP	PCNA1_RFP	0.79 ± 0.05	2.57 ± 0.01	2.54 ± 0.02	1.17
CycD2;1_GFP	PCNA2_RFP	0.77 ± 0.04	2.57 ± 0.01	2.54 ± 0.01	0.94
CycD3;1_GFP	PCNA1_RFP	0.90 ± 0.00	2.52 ± 0.01	2.48 ± 0.01	1.75
CycD3;1_GFP	PCNA2_RFP	0.89 ± 0.01	2.52 ± 0.01	2.50 ± 0.01	0.87
CycD3;2_GFP	PCNA1_RFP	0.82 ± 0.03	2.51 ± 0.01	2.49 ± 0.01	0.72
CycD3;2_GFP	PCNA2_RFP	0.83 ± 0.02	2.51 ± 0.01	2.48 ± 0.01	1.04
CycD3;3_GFP	PCNA1_RFP	0.84 ± 0.01	2.53 ± 0.01	2.49 ± 0.01	1.50
CycD3;3_GFP	PCNA2_RFP	0.86 ± 0.03	2.53 ± 0.01	2.49 ± 0.01	1.42
CycD4;1_GFP	PCNA1_RFP	0.93 ± 0.01	2.54 ± 0.03	2.49 ± 0.01	2.13
CycD4;1_GFP	PCNA2_RFP	0.91 ± 0.01	2.54 ± 0.03	2.50 ± 0.02	1.50
CycD4;2_GFP	PCNA1_RFP	0.86 ± 0.02	2.52 ± 0.02	2.50 ± 0.01	0.48
CycD4;2_GFP	PCNA2_RFP	0.80 ± 0.03	2.52 ± 0.02	2.50 ± 0.02	0.48
CycD5;1_GFP	PCNA1_RFP	0.85 ± 0.01	2.40 ± 0.03	2.39 ± 0.03	0.50
CycD5;1_GFP	PCNA2_RFP	0.86 ± 0.01	2.40 ± 0.03	2.40 ± 0.04	0.08
CycD6;1_GFP	PCNA1_RFP	0.87 ± 0.01	2.49 ± 0.01	2.46 ± 0.02	0.57
CycD6;1_GFP	PCNA2_RFP	0.87 ± 0.02	2.49 ± 0.01	2.48 ± 0.01	0.08
CycD7;1_GFP	PCNA1_RFP	0.92 ± 0.01	2.49 ± 0.02	2.43 ± 0.01	1.94
CycD7;1_GFP	PCNA2_RFP	0.90 ± 0.01	2.49 ± 0.02	2.44 ± 0.02	1.53

*(τ D) and (τ DA) represent the average fluorescence lifetimes of donor proteins and donor proteins in the presence of a potential acceptor respectively. SD, standard deviation. The results are an average of three independent experiments*.

## Discussion

Arabidopsis *PCNA1* and *PCNA2* genes are spatially separated and located on chromosome 1 and 2 respectively (Arabidopsis Genome Initiative, 2000). The high level of identity between Arabidopsis PCNA1 and PCNA2 proteins (97%) results from there being only nine differences in amino acid residues. Although studies on eukaryotic PCNA have been carried out over the last decades, the question of the functional relevance of PCNA1/2 proteins in plants is still a matter for debate. Crystal structure analysis of Arabidopsis PCNA (Strzalka et al., 2009) does not indicate any functional differentiation between PCNA1 and PCNA2. However, results presented by others suggest a peculiar role for just PCNA2 in DNA repair (Anderson et al., 2008; Amoroso et al., 2011).

The *in silico* study of Arabidopsis D-type cyclins revealed that the motif/domain organization of individual proteins is not identical to corresponding maize cyclins. In Arabidopsis the pRB binding motif (LXCXE) was not detected in CycD4;2, CycD5;1, and CycD6;1 (Figure 1) while in case of maize proteins it is absent only in the CycD6;1 (Buendia-Monreal et al., 2011). Testing Arabidopsis proteins using Pfam database we could not detect the C-terminal domain in the CycD4;1 (splicing variant AT5G65420.2) and CycD5;1 (both splicing variants). Similar analysis for maize D-type cyclins showed that C-terminal domain was not found in the CycD3;1a, CycD3;1b, CycD5;3a, CycD5;3b, and also CycD7;1 (Buendia-Monreal et al., 2011). Investigation of genes structure demonstrated that Arabidopsis CycD1;1/CycD2;1 (both splicing variants)/CycD4;1 (splicing variant AT5G65420.1)/CycD4;2/CycD6;1 and CycD5;1 are products of six and five exons respectively (Figure 1) similarly to the corresponding maize transcripts (Buendia-Monreal et al., 2011). On the other hand the number of exons identified for the other corresponding Arabidopsis/maize D-type cyclin transcripts differed. The *in silico* investigation, where the presence of the putative nuclear localization signal in D-type cyclins was analyzed, suggests that the mechanism of CycD2;1, CycD4;1, CycD4;2, and CycD7;1 (Figure 1) import into the nucleus might be not dependent on importin α.

To investigate whether Arabidopsis PCNA1/2 can play different functions in cell cycle control we tested the formation of complexes between PCNA1/2 and D-type cyclins. This group of proteins was selected based on previous data from maize and animal studies where PCNA was demonstrated to interact/co-precipitate with D-type cyclins (Matsuoka et al., 1994; Shimizu and Mori, 1998; Gutierrez et al., 2005; Lara-Nunez et al., 2008; Becerril et al., 2012). Firstly, we tested the subcellular localization of Arabidopsis D-type cyclins to confirm that they are present in the same compartment as PCNA1 and PCNA2 (Strzalka et al., 2012, 2013). All of the tested cyclins could be observed in the nucleus, although not all of them were detected in the cytoplasm (Figure 2). Most of our results were in accordance with previous reports with a few exceptions (Kono et al., 2007; Boruc et al., 2010). In our experimental conditions CycD2;1 was observed in the nucleus and cytoplasm which is in opposition to the results of others who showed exclusively nuclear localization of this protein (Boruc et al., 2010; Sanz et al., 2011). In contrast to data published by Boruc and co-workers our studies showed only nuclear localization of CycD3;1 (Boruc et al., 2010). The analysis of CycD3;3 (this study) revealed that this protein was observed only in the nucleus. This is in contrast to data presented for tobacco CycD3;3 which was detected primarily in the nucleus although it was also visible in the cytoplasm (Nakagami et al., 2002). Previous studies of CycD6;1 subcellular localization in the root cells revealed that in some cells it could be detected in the nucleus, as in our studies, and in other cells in the cytoplasm (Cruz-Ramırez et al., 2012). To conclude, in this study we found some discrepancies in the subcellular localization of the tested cyclins when compared to data presented previously in other reports. This may results from, e.g., (i) different tissue type, (ii) type of expression system (transient/stable, Arabidopsis/tobacco), and (iii) promotor type (35S/natural) used during subcellular localization analysis of these proteins. Taking into account the fact that in the nucleus we could detect all of the analyzed Arabidopsis D-type cyclins, we tested their ability to form complexes with either PCNA1 or PCNA2. Our experiments revealed that among all the cyclins tested only CycD1;1, CycD3;1, and CycD3;3 could not be detected in complexes with PCNA1/2 by any of used experimental technique. This might be due to several reasons: (i) these cyclins are not involved in PCNA-dependent cell cycle control, (ii) there are other factor(s), absent under our experimental conditions, which are necessary for complex formation, and (iii) steric hindrance may prevent complex formation.

The experimental results from the Y2H system and BiFC showed the presence of complex composed of CycD2;1 and PCNA1/2. This is in agreement with data from studies, conducted on maize embryo axes, where PCNA was shown to co-precipitate with CycD2 (Gutierrez et al., 2005). The results from our FLIM-FRET analysis did not indicated that CycD2;1 and PCNA1 are parts of the same complex. The calculated FRET efficiency was less than 2% which is close to the value calculated for the non-interacting GFP/RFP pair. Moreover, neither did our FLIM-FRET analyzes demonstrate the presence of CycD2;1/PCNA2 complexes in tobacco leaf cells. This could be a result of GFP/RFP steric hindrance. The results of the CycD3;2 study in the Y2H system, which are opposite to the data from the BiFC and FLIM-FRET analyzes, suggested that Arabidopsis D3-type cyclins can form complexes with PCNA1/2. This is consistent with other data presented by Shimizu and Mori, who showed presence of the pea CycD3;1/PCNA complex (Shimizu and Mori, 1998). Moreover, the authors using anti-CycD3;l immunoaffinity column chromatography demonstrated that PCNA could be co-precipitated only when extracts were prepared from dormant buds, but not growing buds. This finding indicates that the formation of D-type cyclin/PCNA complexes in plant cells might be dependent on cell status/developmental stage which could explain our results from the CycD3;2/PCNA analyzes. Studying CycD4;1 and CycD4;2, using Y2H, BiFC and FLIM-FRET technique, we discovered that both proteins were able to form complexes with PCNA1 and PCNA2. This result is consistent with experiments performed on maize embryos where in immunoprecipitation studies PCNA was shown to be associated with CycD4;1 and CycD4;2 (Lara-Nunez et al., 2008; Becerril et al., 2012). Unexpectedly, the BiFC findings demonstrated that the complex of CycD4;1 and PCNA1/2 was not localized in the nucleus and cytoplasm, as observed for these proteins when analyzed separately, but was exclusively in the nuclear compartment (Figure 2; Strzalka et al., 2012). This suggests that the formation of the CycD4;1/PCNA complex *in planta* is dependent on nuclear factor(s) or post-translational modification(s) which have not yet been identified. CycD5;1 studies in the yeast system revealed that this protein could be observed only in complex with PCNA2. This may suggest functional differentiation between PCNA1 and PCNA2 in the context of interaction with D-type cyclins. Nevertheless, BiFC analysis did not confirm this finding but revealed that CycD5;1 formed complexes with both PCNA1 and PCNA2. In contrast to the BiFC experiments, the FLIM-FRET analysis did not show the formation of complexes between CycD5;1 and PCNA1/2 which is most probably the result of steric hindrance caused by GFP/RFP proteins. The results of the CycD6;1 analysis were identical to data from the CycD4;2 studies. Finally, in contrast to the results from Y2H analysis of CycD7;1, a very poorly characterized Arabidopsis D-type cyclin, the data from BiFC and FLIM-FRET studies showed formation of putative CycD7;1/PCNA1 or CycD7;1/PCNA2 complexes exclusively in the nucleus. Comparative analysis of the results from the FLIM-FRET analysis undoubtedly showed that the site of RFP fusion to PCNA affected the possibility of PCNA/D-type cyclin complexes formation.

The differences in the FRET efficiency detected for individual CycD and PCNA1/2 complexes were unexpected and possibly result from differences between PCNA1 and PCNA2 amino acid sequences. Unfortunately, the biochemical data which could help to characterize and compare the properties of Arabidopsis CycD and PCNA1/2 complexes are still not available mainly due to the lack of an efficient system which could provide large quantities of biologically active D-type cyclins. These data are necessary to verify and understand in detail the impact of PCNA1/2 amino acid differences on the formation of complexes with individual D-type cyclines, in the absence of other nuclear proteins.

In conclusion, we showed that most of the tested Arabidopsis D-type cyclins formed complexes with PCNA1/2 under the experimental conditions applied. The data from the Y2H and BiFC experiments did not provide convincing evidence for functional differentiation between PCNA1 and PCNA2 proteins in the context of their interaction with Arabidopsis D-type cyclins. However, FLIM-FRET analysis revealed a significant difference in the distance between PCNA1/2 and the CycD4;1, CycD4;2, CycD6;1, or CycD7;1 proteins (Table 1). The nine amino acid differences between PCNA1 and PCNA2 seems to have an impact on the architecture of CycD/PCNA complexes, which is reflected in the different spatial proximity between PCNA1/CycD4;1, PCNA1/CycD6;1, PCNA1/CycD7;1, and PCNA2/CycD4;2 proteins in comparison to PCNA2/CycD4;1, PCNA2/CycD6;1, PCNA2/CycD7;1, and PCNA1/CycD4;2 respectively. The key question is how far these differences are functionally relevant? It should be verified in the future whether the difference in the distance between PCNA1/2 and CycDs is related to the difference in the affinity between individual CycDs and PCNA1/PCNA2. If so, this could suggest that the formation of, e.g., CycD4;1/PCNA1 complex, which is characterized by a shorter distance between this protein pair and possibly a lower value of the dissociation constant when compared to CycD4;1/PCNA2, could be preferred. If this is true then the complex characterized by a higher association constant could be functionally relevant. However, to verify this hypothesis additional new data are needed. The presented study does not yet provide the evidence for functional differentiation between PCNA1/CycD and PCNA2/CycD complexes in Arabidopsis. On the other hand the fact that not all of the 10 tested D-type cyclins were observed in complexes with PCNA shows that their roles are not equal in the context of interactions with Arabidopsis PCNA1/2. To conclude, the presented data are one of the significant milestones before the functional relevance of identified Arabidopsis CycDs/PCNA complexes will be the finally uncovered, especially in DNA replication and cell cycle control.

### Conflict of interest statement

The authors declare that the research was conducted in the absence of any commercial or financial relationships that could be construed as a potential conflict of interest.

## Supplementary material

Supplementary Figure 1 **(A, B)** Analysis of split GFP complexes formed between PCNA1 or PCNA2 and D-type cyclins. Confocal images of *N. benthamiana* leaf cells transiently expressing the analyzed open reading frames. All the images are overlays of bright field, autofluorescence of chlorophyll (red) and GFP fluorescence (green). This result is representative of three independently repeated experiments. **(C)** Analysis of D-type cyclin subcellular localization and the formation of complexes with either PCNA1 or PCNA2—bright-field confocal images of *N. benthamiana* leaf cells transiently expressing open reading frames presented at Figure 2. **(D)** Analysis of Arabidopsis PCNA1_GFP and PCNA2_GFP subcellular localization. The upper panel represents images that are overlays of bright field, autofluorescence of chlorophyll (red) and GFP fluorescence (green). The lower panel represents bright field confocal images of *N. benthamiana* leaf cells transiently expressing PCNA1_GFP or PCNA2_GFP.

Supplementary Table 1 **List of primers used in this study**.

Supplementary Table 2 **The list of plasmids used in this study**.
