# Supplementary figures and images for: Arabidopsis PCNAs form complexes with selected D-type cyclins
Source: Front Plant Sci. 2015 Jul 17;6:516. doi: 10.3389/fpls.2015.00516 (PMC4550699; doi:10.3389/fpls.2015.00516)

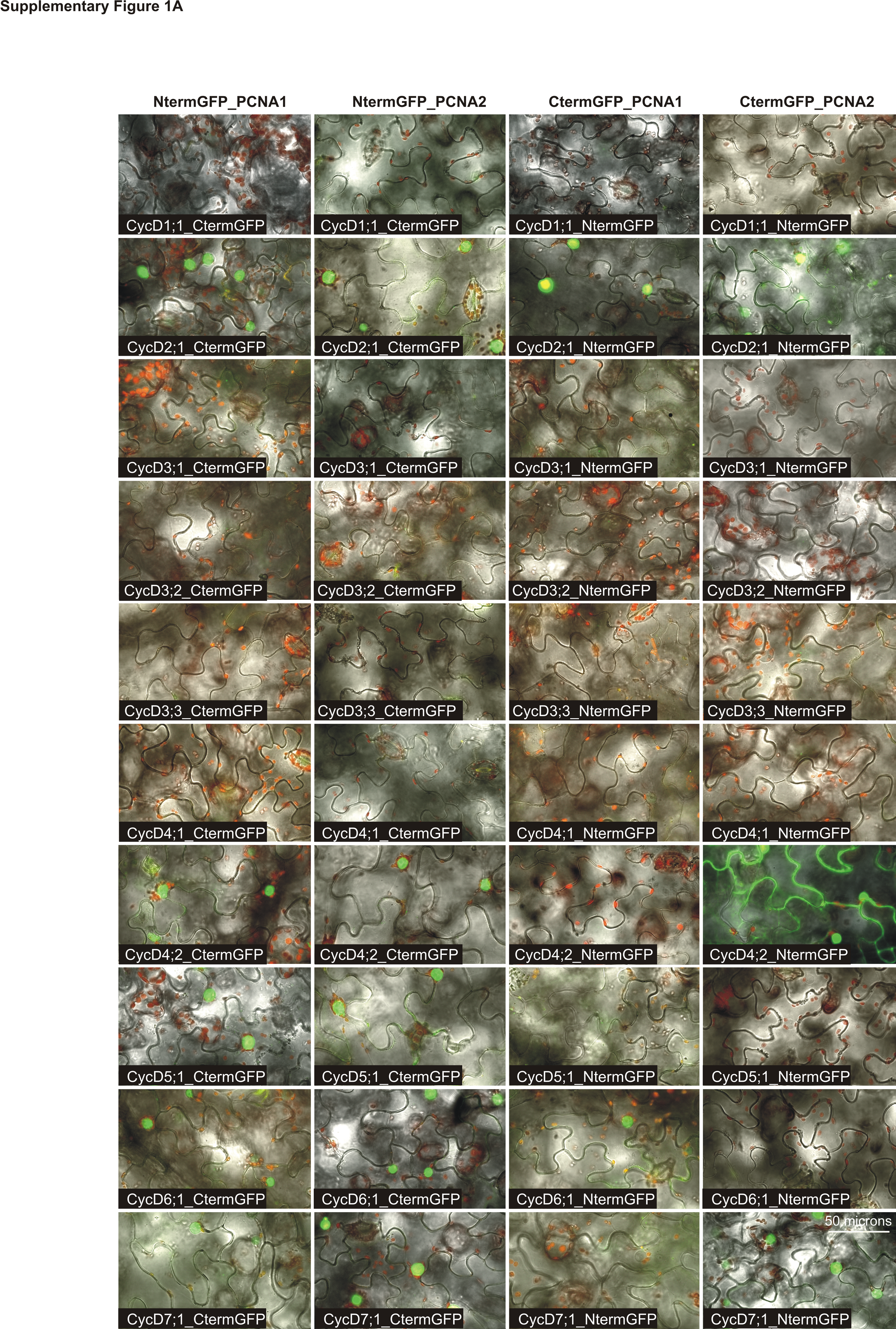

Supplement: Supplementary Figure 1 — (A, B) Analysis of split GFP complexes formed between PCNA1 or PCNA2 and D-type cyclins. Confocal images of N. benthamiana leaf cells transiently expressing the analyzed open reading frames. All the images are overlays of bright field, autofluorescence of chlorophyll (red) and GFP fluorescence (green). This result is representative of three independently repeated experiments. (C) Analysis of D-type cyclin subcellular localization and the formation of complexes with either PCNA1 or PCNA2—bright-field confocal images of N. benthamiana leaf cells transiently expressing open reading frames presented at Figure 2. (D) Analysis of Arabidopsis PCNA1_GFP and PCNA2_GFP subcellular localization. The upper panel represents images that are overlays of bright field, autofluorescence of chlorophyll (red) and GFP fluorescence (green). The lower panel represents bright field confocal images of N. benthamiana leaf cells transiently expressing PCNA1_GFP or PCNA2_GFP. [file Image1.TIF]

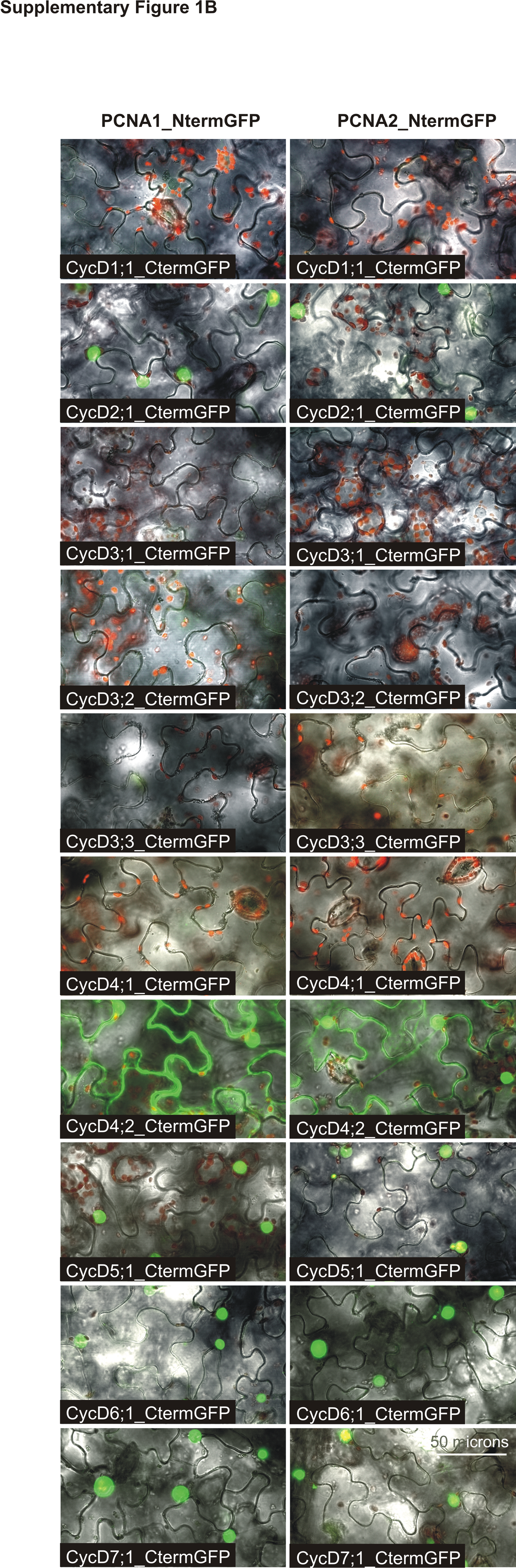

Supplement: Supplementary file 3 [file Image2.TIF]

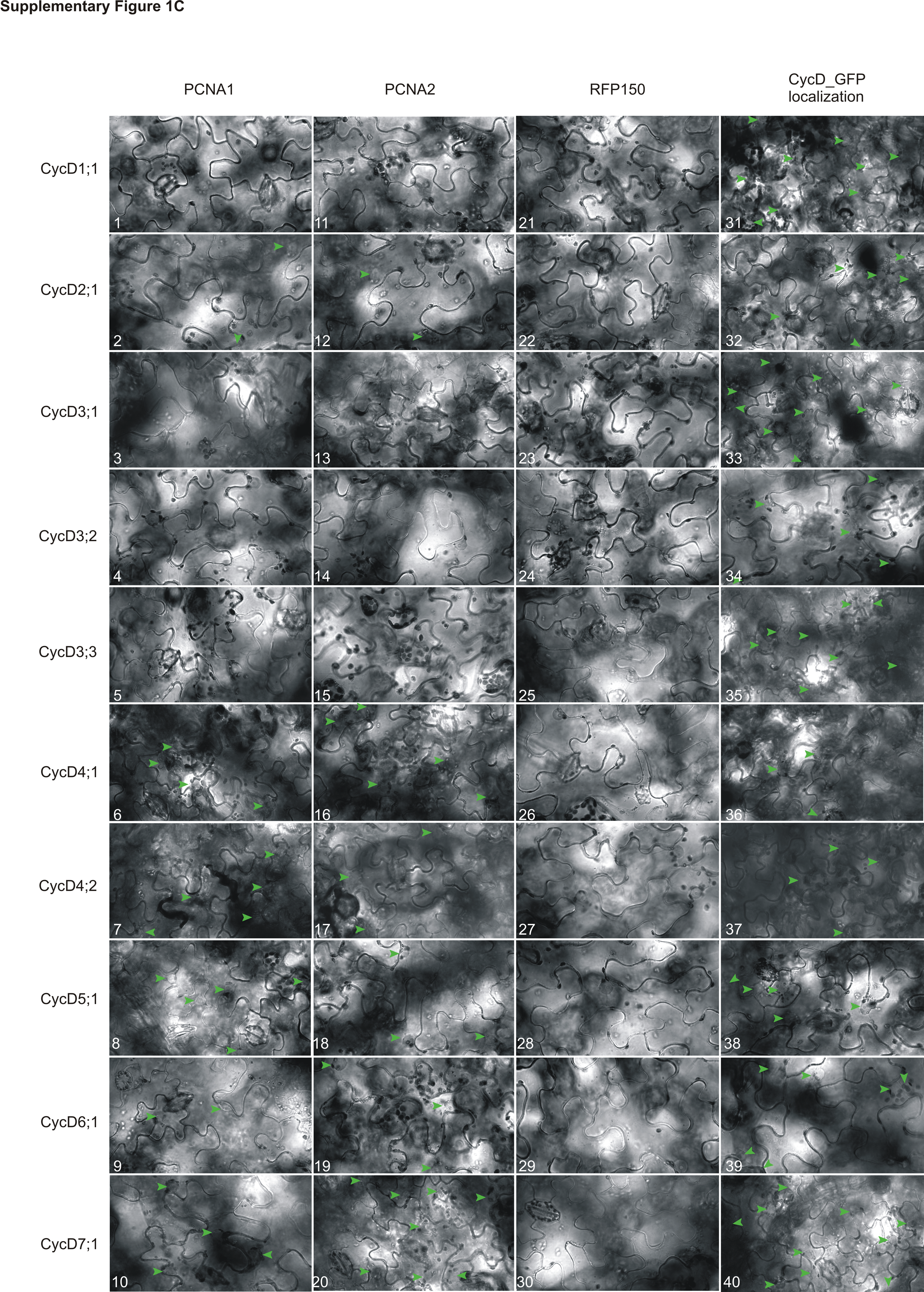

Supplement: Supplementary file 4 [file Image3.TIF]

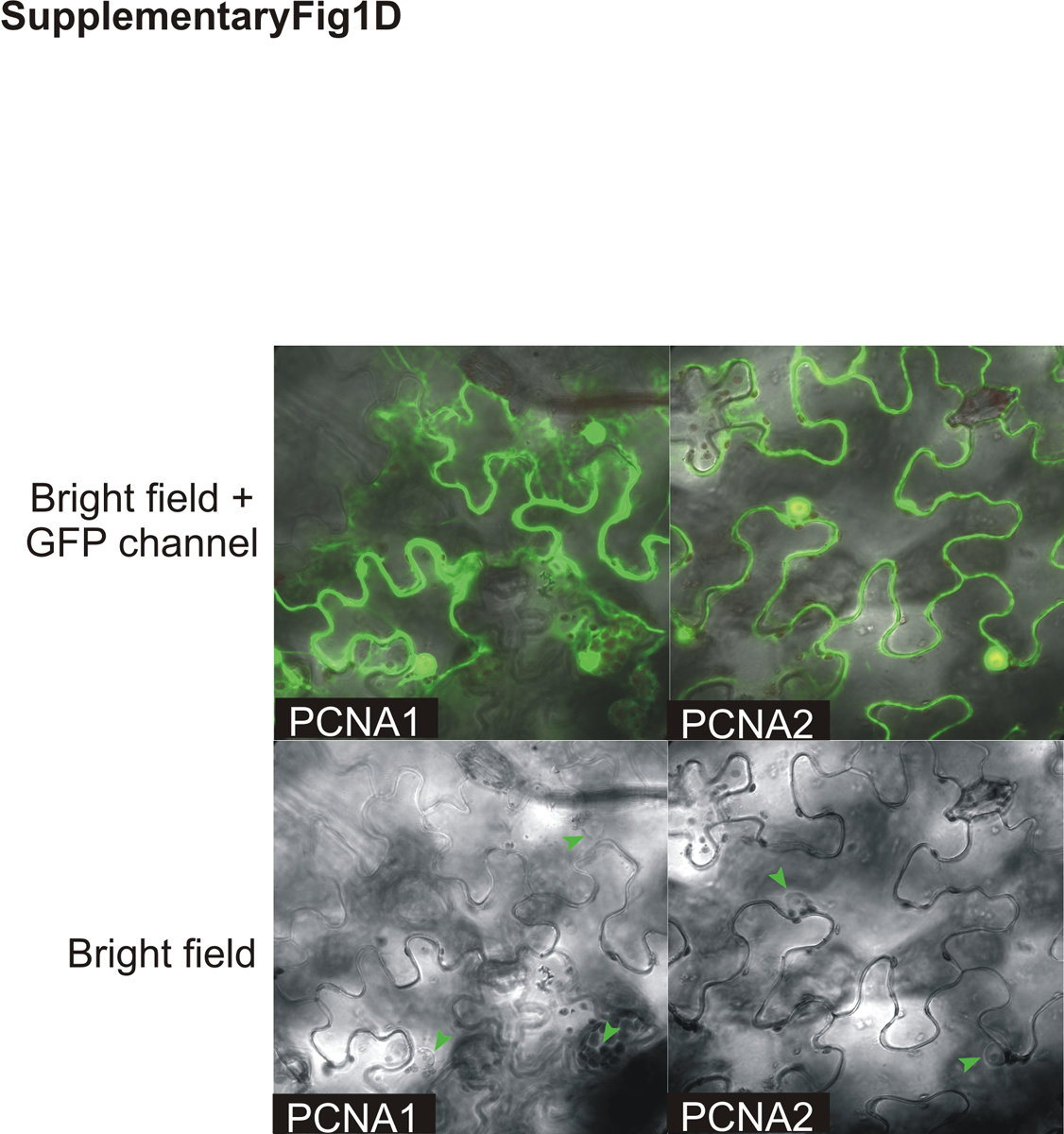

Supplement: Supplementary file 5 [file Image4.TIF]
